# Quality Multiverse of Beef and Pork Meat in a Single Score

**DOI:** 10.3390/foods11081154

**Published:** 2022-04-15

**Authors:** Sara Rajic, Stefan Simunovic, Vesna Djordjevic, Mladen Raseta, Igor Tomasevic, Ilija Djekic

**Affiliations:** 1 Institute of Meat Hygiene and Technology, Kaćanskog 13, 11040 Belgrade, Serbia; stefan.simunovic@inmes.rs (S.S.); vesna.djordjevic@inmes.rs (V.D.); mladen.raseta@inmes.rs (M.R.); 2 Department of Food Safety and Quality Management, Faculty of Agriculture, University of Belgrade, Nemanjina 6, 11080 Belgrade, Serbia; idjekic@agrif.bg.ac.rs; 3 Department of Animal Source Food Technology, Faculty of Agriculture, University of Belgrade, Nemanjina 6, 11080 Belgrade, Serbia; tbigor@agrif.bg.ac.rs

**Keywords:** meat quality, intrinsic attributes, extrinsic attributes, quality wheel, total quality index

## Abstract

The rationale behind this review is the potential of developing a single score tool for meat quality evaluation based on visual and sensorial assessments of fresh meat. Based on the known sensory wheel concept, the first step was to create quality wheels capturing most common intrinsic and extrinsic quality cues of pork and beef outlined in the latest scientific papers. This resulted in identifying meat color, sensory characteristics and fat content as the most important intrinsic quality cues of fresh beef and pork. Furthermore, the highest number of studies showed the importance of price, certification logos and brand for beef quality evaluation. According to recent articles, price, breed, animal welfare and a veterinary certificate are the most important extrinsic attributes for pork consumers. The second step was to develop a single-score tool named the “Meat quality index”. It has been developed in line with published approaches of different total quality index concepts used in the food sector, providing insights into its application in the meat sector. As a result, this review proposes a unique approach in using quality index application, through the consumer’s preferences aspect of fresh meat.

## 1. Introduction

The production of meat according to the technical product specifications helps producers to meet consumers’ demands for the most common quality characteristics of meat, which generally encompass microbiological attributes, chemical attributes (fat, protein and moisture) and sensory attributes (color, tenderness and flavor), as well as other attributes (breed, type of package and expiration date). However, the evolution of the consumer’s perception, expectations and needs places additional quality criteria for meat producers that they need to fulfill [1]. Taking into account meat quality perception before and after beef consumption, two dimensions have been raised: expected and experienced quality. Expected quality is formed at the point of sale based on available quality cues, while experienced quality arises at the point of consumption, mainly based on sensory perception.

In recent years, consumers developed demanding requirements in terms of meat quality, which are linked with their preferences affected by many diverse factors. By analyzing these factors, meat producers can properly react in order to offer diversified meat products to consumers. Therefore, the first challenge is to identify quality cues currently used by consumers to evaluate meat. These quality cues are usually divided into intrinsic and extrinsic characteristics [2]. The key distinction between intrinsic and extrinsic quality cues is that intrinsic quality cues are part of the physical product. Thus, intrinsic attributes such are meat color, cut, fat, marbling, amount of drip and texture can be directly detected at the point of purchase by consumers. On the other side, when it comes to extrinsic quality cues, consumers must be informed about them through the label or through advertising. These characteristics, such as price, promotion, brand name, package, storage temperature, certification logos and so on, are related to the product but are not physically part of it.

Some authors have studied the relative importance of meat quality attributes from a consumer’s perspective, by comparing these attributes, like in the case of beef, by analyzing their interrelationships, the influence of cues on expected quality and the correlation between extrinsic and intrinsic quality cues [1,3,4,5,6]. The outcome of these studies was a clear categorization of different intrinsic and extrinsic quality attributes.

Sensory science has developed a sensory wheel to visually depict different sensations that can possibly be associated with a food product during consumption [7]. In that sense, the development of a quality wheel (QW) would be to facilitate the process of meat purchasing by consumers. Therefore, these wheels can address the needs of consumers during meat purchasing, but they also serve as an aid to manufacturers during their final inspection of meat [8]. Based on known intrinsic and extrinsic quality cues, it is expected that QW can enable easier identification of desirable cues.

When it comes to quality indexes, this approach can be found in the food sector mainly in papers analyzing nutrition (diet quality index) and different types of quality indexes related to the evaluation of food quality. One of the first food quality indexes was developed with the aim to make a model that could enable overall description of food quality [9]. The index was based on a weighted sum of individual quality parameters from 0 to 1, with 0 representing the worst and 1 the best food quality. Furthermore, a more sophisticated model was proposed, considering nine parameters used for the overall quality evaluation of extra-virgin olive oil [10]. In a similar manner, other authors explored and verified these indexes for evaluating the quality of different products such as bread [11], cocoa [12], mushrooms [13,14], juices [15] and dried apples [16]. To date, quality indexes related to meat have not been investigated.

The main objective of this paper was to consider the potentials for developing QWs based on recent scientific papers related to intrinsic and extrinsic quality attributes of beef and pork meat that pave the way for creating a unique total quality index (QI). 

## 2. Research Methodology

A literature review has been performed to identify the main intrinsic and extrinsic characteristics associated with beef and pork meat. To perform this task, the following criteria were applied: range of years (2018–2021); type of articles (research and review); type of journals (only with impact factor); search engine (Google Scholar); keywords used (“intrinsic and extrinsic quality characteristics of beef“) (Table 1).

**Figure 1:**
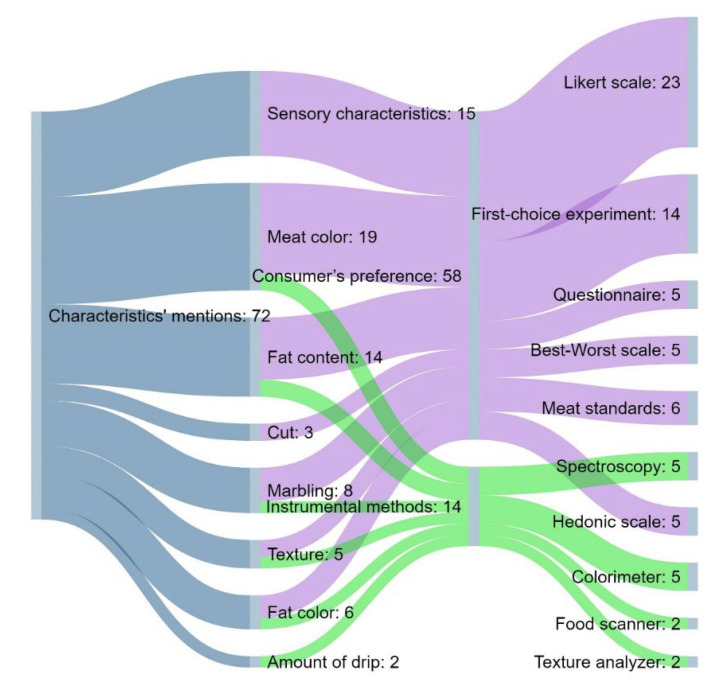
Sankey chart showing the distribution of the types of methods per analyzed intrinsic characteristics of beef quality.

**Figure 2:**
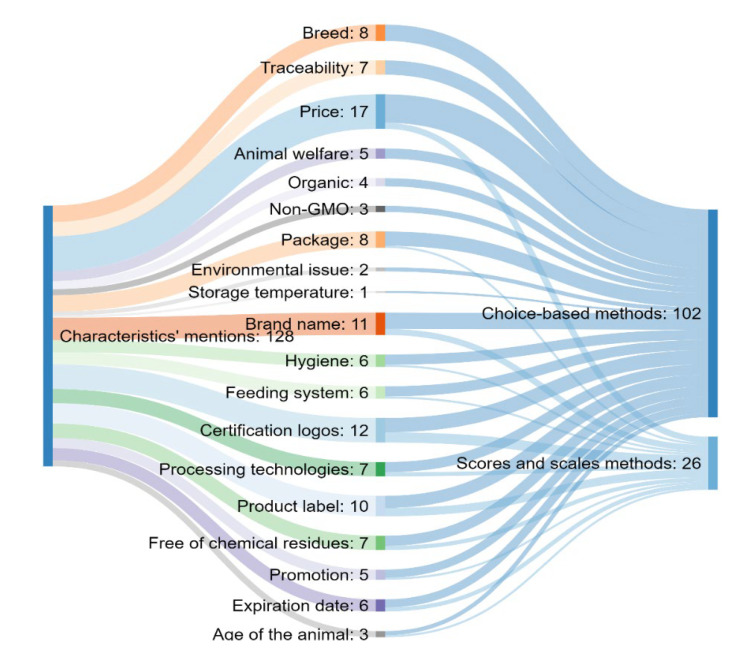
Sankey chart showing the corresponding groups of methods for each extrinsic characteristic of beef quality.

At this phase, the authors explored how many papers studied beef quality using the concepts of intrinsic and extrinsic characteristics. The second step was to place the consumer preference context. The final phase was to exclude papers that were not relevant for this review. The final selection revealed 41 studies that suggested different intrinsic and extrinsic cues. The intrinsic characteristics of beef quality are presented in Figure 1, while Figure 2 depicts the extrinsic characteristics of beef quality. In the highest number of papers, odor, tenderness, juiciness, flavor and overall liking were examined as sensory characteristics. Results from this search were used as a basis for developing quality wheels.

The assessment of consumer responses on beef attributes was predominantly obtained by the use of a five-point Likert scale. The second consumer’s preference method frequently used was the first-choice experiment, which encourages consumers to select among different product profiles (choice scenario). Many authors use standards to present the levels for attributes in their experiment’s designs. Thus, the use of a standard was included in the summary of consumer’s preference-methods. However, in the sample of articles used in this study, the Australian meat standard was predominantly used [17]. On the other side, scale-based methods present useful tools for assessing consumer’s responses. In that sense, the Likert scale was mainly used (Figure 1). The considerable number of studies proposed near-infrared spectroscopy (NIRS) as an instrumental method to predict beef consumers’ perceptions [18]. As presented in Figure 1, the use of a colorimeter has a high rate in the instrumental evaluation of beef intrinsic quality cues, where increased use of a Minolta CM-600d spectrophotometer was noticed [19,20]. Other instrumental methods used for beef quality evaluation were the use of a food scanner for the analysis of protein, fat and moisture in meat and meat products and use of a texture analyzer for the analysis of meat toughness, which was measured by the Warner–Bratzler (WB) shear test [18].

Choice-based methods have been noted as the most practical methods for evaluating extrinsic beef quality. These characteristics also became subjects of ranking or scaling methods, mostly in cases of evaluating their importance by consumers (Figure 2). Concerning choice-based methods, discrete choice experiments and questionnaires took leading positions, while visual appraisal was the least frequently used method. However, in comparison with scale-based methods, choice-based methods were more often chosen to evaluate consumers’ preferences among extrinsic quality cues.

The same approach was applied for analyzing “intrinsic and extrinsic quality characteristics of pork consumer preference“ (Table 2).

**Figure 3:**
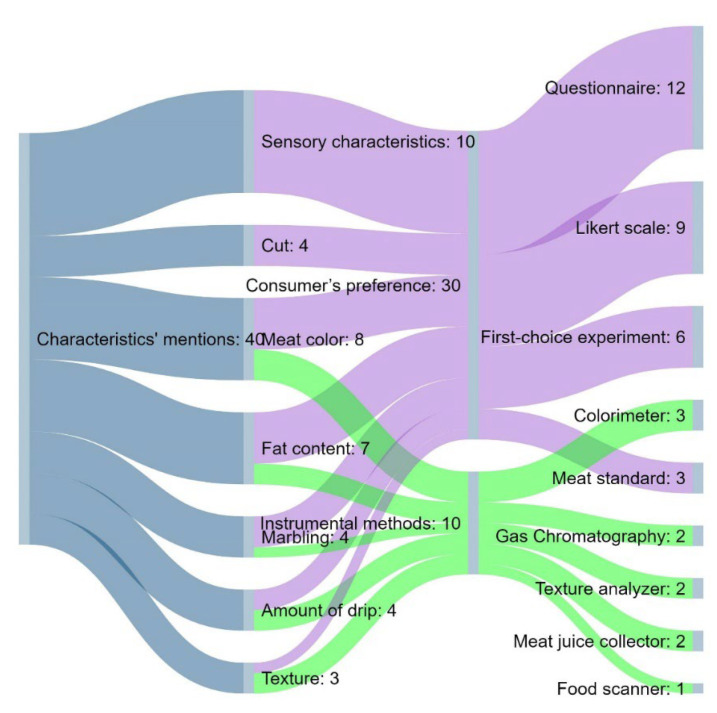
Sankey chart showing the distribution of the types of methods per analyzed intrinsic characteristics of pork quality.

The most dominant instrumental method for evaluating pork intrinsic characteristics related to color was the use of a colorimeter or computer vision system [21]. When it comes to consumer’s preference-methods, in comparison with consumer’s preference-methods for evaluating the quality of beef, standards such as the NPPC (National Pork Producers Council) Pork Quality Standards were less used for evaluating pork quality compared to beef standards. Within the analyzed papers on pork quality, a Likert scale and the first-choice experiment were less employed in exploring the consumers’ preferences than in the case of beef. A questionnaire was revealed to be one of the main tools for pork consumers’ preference evaluation (Figure 3). The consumer’s responses to sensory characteristics were mostly recorded using the questionnaire format. Furthermore, consumers were mainly asked to evaluate the odor, tenderness, juiciness, flavor and overall appearance.

**Figure 4:**
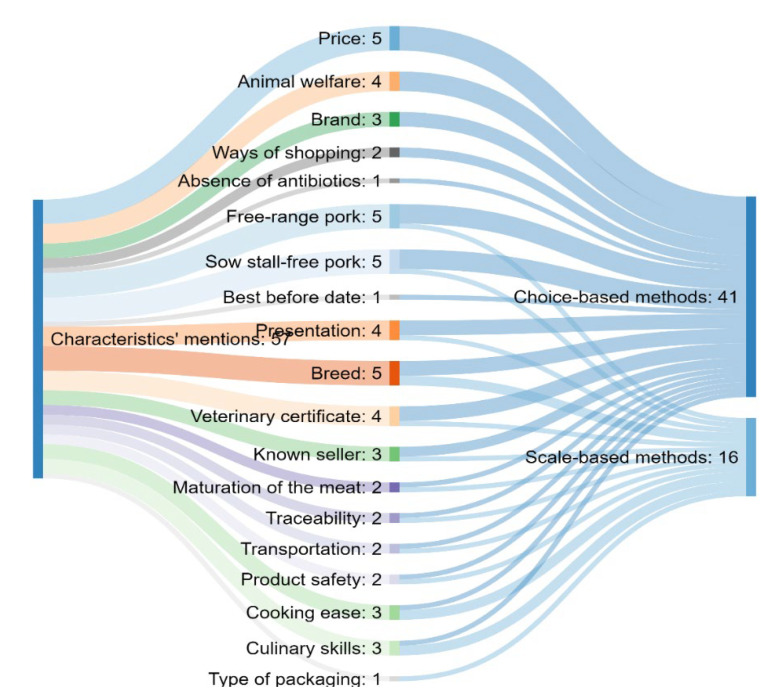
Sankey chart showing the corresponding groups of methods for each extrinsic characteristic of pork quality.

It was revealed that the choice-based methods were more frequently used to measure the importance of extrinsic attributes of pork quality (Figure 4).

## 3. Quality Cues

### 3.1. Intrinsic Quality Cues

Basic intrinsic attributes of beef are meat color, fat content and cut [4], but other important characteristics are fat marbling, amount of drip, texture, freshness, juiciness, tenderness, flavor and taste [22,23]. Some authors emphasize an added amount of subcutaneous fat, consistency and overall appearance as important attributes [24]. All intrinsic characteristics can be categorized as search, experience and credence [2]. Search attributes refer to these which can be evaluated at the point of purchase, such as meat color, cut, fat marbling, etc. The experience attributes are the ones which cannot be assessed prior to consumption. Finally, credence attributes are associated with health and process benefits where consumers rely on the information provided by producers or independent institutions for process/product certification [2].

#### 3.1.1. Meat Color

Meat color correlates with myoglobin content, but it also is closely related to intramuscular fat content and pH [25]. Furthermore, other post-harvest factors affecting the color of fresh meat are the temperature, package conditions and lipid oxidation during aging and exposure to consumers. On the other side, the color intensity of meat is determined by pre-harvest factors such as species, stress, sex, age of animal and animal diet (including feed withdrawal time and the type of animal feed) [26]. This was identified as the most important intrinsic characteristic for consumers based on its occurrence in the largest number of studies (Figure 1). Furthermore, the consumers’ ability to discern between systematically varied colors of meat was developed [27].

#### 3.1.2. Sensory Characteristics

It was mentioned that odor, tenderness, juiciness, flavor and overall liking were most frequently analyzed through sensory testing of fresh meat. Sensory evaluation serves the meat industry and scientists to quantify the tenderness of meat from actual consumer feedback [28]. The nutritional value of meat and healthiness became important motivators for purchasing fresh meat, due to the increased consumers’ awareness of the relationships between diet and health [29].

#### 3.1.3. Fat Content

Fat content is noted as one of the main factors when determining meat suitability [30]. From a physiological point of view, fats are important as they contain a number of vitamins and essential fatty acids and present an important source of energy. Furthermore, fat contributes to different sensory characteristics of flavor, juiciness, appearance and tenderness. However, an interesting trend occurs where consumers more often choose leaner meat and evaluate leanness as an important attribute [31]. Hence, when a product has a highlighted attribute of “low fat”, its price increased compared to products that did not highlight this attribute [32]. However, the applicability of fat content as a determinant of meat quality is most frequently presented through consumers’ responses on Likert scales [23]. Furthermore, the development of instrumental methods for evaluating fat content in meat and meat products is inevitable [33,34]. 

In this study, meat color, sensory characteristics and fat content were represented as the most important cues of fresh meat quality, both for beef and pork, since they were examined in the highest number of analyzed papers (Figure 1 and Figure 3).

#### 3.1.4. Marbling

It has been generally accepted that a certain degree of marbling has a positive effect on the juiciness, tenderness, palatability and flavor of meat [35]. Furthermore, marbling is often considered as an important characteristic that affects a consumer’s purchase decisions [36]. A consumer’s concerns about marbling and subcutaneous fat content prompted meat industries to start using standards’ grading systems. Furthermore, marbling has been included as one of the main determinants in the beef quality grading system [17]. The development of marbling changes the solubility and amount of intramuscular connective tissue, which positively impacts tenderness [37].

#### 3.1.5. Cut

The size of a primal cut (initially separated from the carcass of an animal during butchering) depends mostly on the size of an animal. All possible beef cuts are defined and described in the Institutional Meat Purchase Specifications and Handbook of Australian Meat 7th Edition [15,38]. Although the uniformity of cut can be roughly defined as the consistency in shape and size of all cuts in one package or at one butcher shelf, this attribute needs to be better explained to consumers [39].

#### 3.1.6. Amount of Drip

Generally, the term drip loss can be defined as the fluid, mainly consisting of water and proteins. The amount of drip or drip loss is usually experimentally measured through the water-holding capacity (WHC) and determined in duplicate on 50 g fresh samples taken at 24 h post-mortem and placed in a special container (meat juice collector). A poor water-holding capacity is unwanted, as it means weight loss. However, the amount of drip of a meat cut depends on the conditions under which it is determined [40].

These conditions are:

- The time postmortem and the duration of measurement;
- Geometry of the piece;
- Temperature during the measurement;
- Type of package;
- The sample’s position within the package.

From the consumer’s point of view, this attribute is directly related to product appearance. According to previous literature, consumers’ choices were mostly based on three appearance characteristics: color, drip loss and fat content [41].

#### 3.1.7. Texture

Meat texture is a multi-parameter attribute that is closely correlated with a sensory evaluation of tenderness. It is possible to improve the beef’s tenderness (texture) by considering factors such as the animal breed and feeding system and post mortem factors, such as carcass refrigeration after slaughter, hot carcass hanging, ageing time and culinary methods [42,43]. The most common instrumental objective tenderness measurement is the Warner–Bratzler shear force.

### 3.2. Extrinsic Quality Cues

The process of evaluating overall quality of fresh meat consists evaluations of both, intrinsic and extrinsic quality cues. Extrinsic quality cues represent information related to the product, its promotion, storage conditions, price, package, etc. The main role of these cues is to influence the consumer’s first impression. 

#### 3.2.1. Brand

The meat brand name is often a synonym for a traceable, guaranteed and authentic product. Although, in the past, fresh meat was mainly unbranded and purchased in butcher shops, nowadays meat producers try to differentiate their products on the basis of branding, especially when brand is connected with products of specific geographical origins and nutritional characteristics. Hence, the brand name is common to the “value added” group of attributes that increase the value of product [44]. As the growth of branded beef sales has been detected, it is important to discover the characteristics of consumers purchasing branded beef products. Hence, Bernues et al. [5] concluded that consumers living in cities paid more attention to the label or brand to get information about the quality of meat. Furthermore, consumers are willing to pay more for branded beef, guaranteed traceability and tastier meat [45].

#### 3.2.2. Label

When it comes to label, it presents an important source of information about quality for consumers concerned about safety and nutrition/health. However, Indonesian consumers preferred not to buy beef with an unclear Halal label in spite of its freshness and red color [46]. Furthermore, Brazilian beef buyers considered the traceability information of the label as an important food security indicator [47]. On the other side, the information on the label is less important than the intrinsic characteristics of beef for the acceptance of a new product [48].

#### 3.2.3. Package

As the color of fresh meat is highly influenced by package, this extrinsic quality cue was defined as important [49]. In addition, package has been repeatedly found to be a strong driver for consumers’ food choice [50]. According to Ardeshiri and Rose [51], the most important extrinsic attribute related to product appearance in beef products is the type of package. When it comes to the comparison between trey-packed and vacuum-packed beef, US consumers preferred vacuum-packed, especially in the summer season [36].

#### 3.2.4. Animal Breed

From the farmers’ point of view, the choice of animal breed depends on the geographical area, a history of breeding a specific domestic breed originating in the territory and the type of production (extensive or intensive). However, consumers often link extensive production with traditional breeds, organic meat and free-range livestock production. According to the study by López-Pedrouso et al. [20], the strongest effect on the physicochemical parameters and sensory profile of three Spanish cattle breeds under different livestock production systems and pre-slaughter handling conditions was had by the breed type. A combination of factors such as the breed of the animal and rigor state can affect the quality characteristics of meat [52].

#### 3.2.5. Animal Welfare

Consumers often associate animal welfare with natural, green, organic and eco-friendly animal production process. The production of free-range and sow stall-free pork leads to organic pork that increases consumers’ willingness to pay [53]. Furthermore, many studies confirm the importance of animal welfare as an attribute in the decision-making process of beef purchase [54,55,56,57,58]. In the study by Boito et al. [24], it was found that for consumers with a higher education, the age of the animal had an influence. Furthermore, the feeding system was found to be an important characteristic for consumers who had completed higher education and postgraduate education. The impact of antibiotic-free claims on the willingness to pay had the highest variance for sirloin steak [36].

#### 3.2.6. Price

One of the most frequently studied attributes was the price of meat. This is a very important extrinsic quality cue, as its increase can reduce meat consumption and increase the availability of meat alternatives. It was found that price strongly influences the purchasing decision [58]. Furthermore, a higher price was associated by consumers with a desirable higher quality, and they were willing to pay significantly more for grass-fed beef compared to conventional beef [59].

#### 3.2.7. Other Extrinsic Quality Cues

When it comes to experience attributes such as cooking ease, culinary skills and ways of shopping, their impact on the consumers’ purchasing decision were ranked as intermediate [41]. Furthermore, extrinsic attributes such as the promotion of beef at markets also had a significant influence on the consumers’ preferences besides beef presentation and butcher’s location [30].

Some future trends lead to situations where a known seller or place of purchasing will not be as significant as other quality cues such as the price or food safety certification (traceability system) [60]. In that sense, this paper reveals that the highest number of studies show the importance of price, certification logos and brand name when it comes to beef quality (Figure 2). According to these findings, it can be assumed that consumers are mostly concerned with price, certification logos and brand name on packed beef. On the other side, price, breed, animal welfare and a veterinary certificate were presented as the most important attributes according to the literature on pork quality (Figure 4).

## 4. Quality Wheels

**Figure 5:**
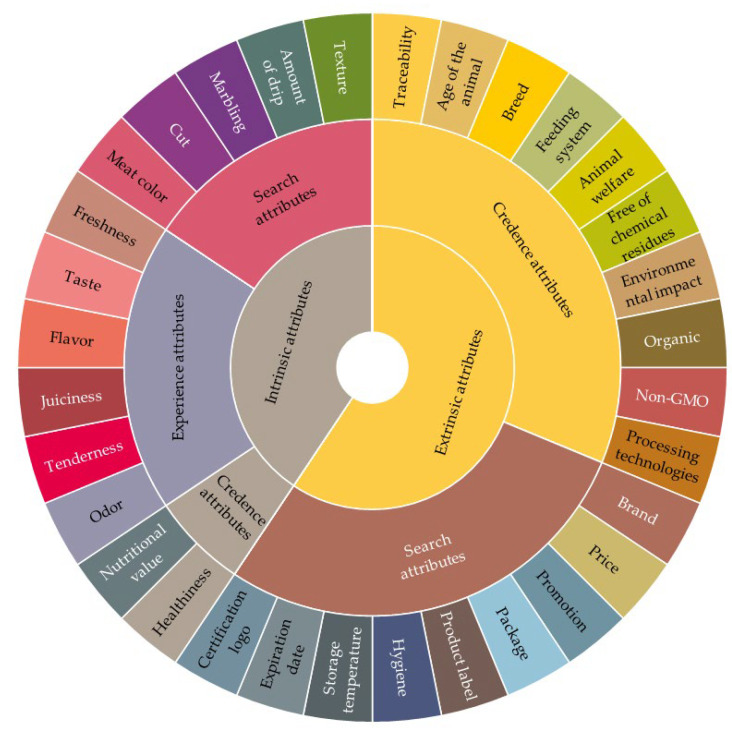
Quality wheel for evaluating beef quality at the point of meat purchase.

**Figure 6:**
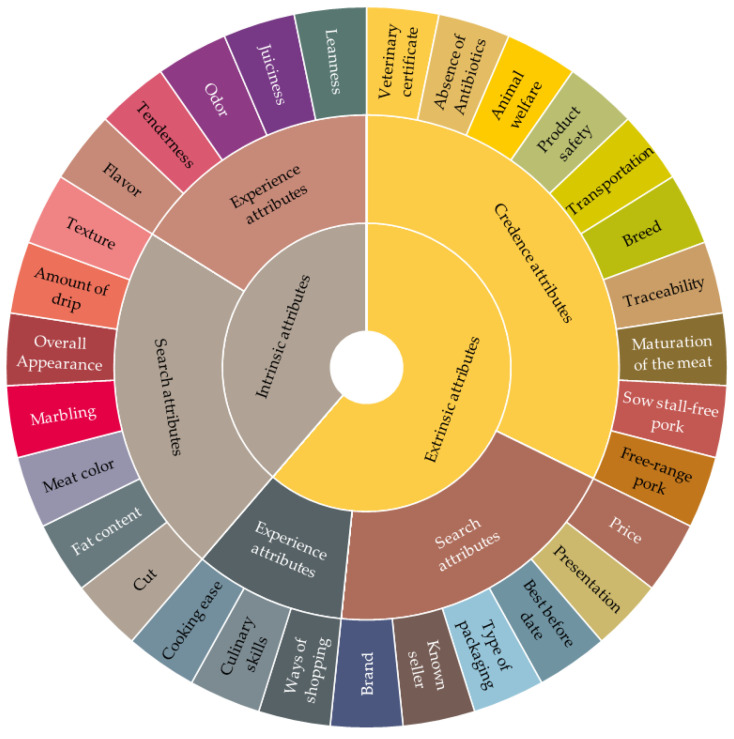
Quality wheel for evaluating pork quality at the point of meat purchase.

The sensory wheel construction techniques were used as a basis for creating suitable QWs to complement the quality evaluation at the point of meat purchase. The concept of creating these tools is similar to the scientific conversion from the sensory lexicon to the sensory wheel. This approach provides an attractive way to convey cues for product differentiation to its potential consumers. Basically, attributes in QWs serve as a checklist against which the attributes of products in front of consumers are compared. A summary of the quality cues provided in the literature review was used for formation of the QWs. The cues that were defined by consumers as the most important were included in beef and pork QWs (Figure 5 and Figure 6).

The quality wheels are dual-purpose tools intended to prevent the lack of communication between meat consumers and producers and help them to clearly understand the quality of meat. As the sensory wheel could be used as the basis of flavor description, the quality wheel could be used as the basis of the description of all of a product’s characteristics. Hence, these two types of wheels function in a similar way. For example, beef flavor wheels guide panelists through more and more precise describing words for flavor, texture and aroma in a direction from the middle to the edge of the wheel. It helps panelists to accurately discover which aroma they feel. Consequently, with a more comprehensive understanding of meat quality, producers can identify directions on how to improve it, while consumers can make better purchase choices.

The principle of using the quality wheel is similar, it helps consumers and producers to find out which quality cues interact with each other. For instance, if consumers seek experienced juiciness, then fat marbling is an important cue for consumers, so they need to consider the type of meat cut. The short loin cuts, such as T-Bone, tenderloin and striploin, and cuts between the 5th and 13th rib are the most preferable cuts where marbling may occur [61,62]. Therefore, the particular level of marbling can depend on the specific animal breed. This is how intrinsic and extrinsic quality cues can be virtually connected through the quality wheel. Furthermore, if consumers look for freshness they should pay attention on meat color, texture and amount of drip. Furthermore, the path of consecutive interconnected quality cues which emerges from the consumers’ search for freshness starts with analyzing meat color, texture and amount of drip at the point of meat purchase. These intrinsic attributes are most closely related to the extrinsic quality cues such as expiration date, storage temperature and package. Afterwards, the extrinsic credence attributes such as processing technologies, animal welfare and transport are highly associated with the mentioned search attributes [31]. The different directions of the cues’ interconnections depend on the awareness and knowledge of both consumers and producers. Thus, these QWs can help end-users to extend their knowledge and develop their awareness of meat quality.

## 5. Quality Index in Meat Industry

As any modification of the production system (technology, package technique, etc.) may affect meat quality and the shelf life of the final product, particular quality and safety evaluations need to be done. In those cases, quality parameters that are under examination could be parameters suitable for future quality index construction. In general, two main questions arise when developing quality indexes. The first is related to the methodology of calculating specific quality attributes, and the second is how to develop an overall single score based on all individual attributes. Based on the work of Finotti et al. [10], individual meat quality indexes are associated with specific meat quality attributes, while the overall (meat) quality index (M_Q_) represents the square root of the sum of squared individual meat quality indexes.

M_Q_ = √∑(X_n_)^2^, (1)

where ∑ summarizes the number of all meat quality attributes and X_n_ presents the individual attribute from a possible range from n = 1 to N attributes, where N presents the total number of attributes studied.

When calculating individual quality indexes, there are three potential rules:

1. The nearer to the target value the parameter is, the better the quality is;
2. The smaller the characteristic’s value is, the better the quality is;
3. The higher the characteristic’s value is, the better the quality is.

One approach in developing an overall meat quality index is to identify key quality attributes (Table 3).

However, for easier understanding of how these rules correspond to meat quality evaluation, the categorization of the rules is outlined in Table 4 andTable 5. The first selection of characteristics should be supported by some previous literature, then be evaluated by consumers in order to find out which characteristics are important. This step serves authors to discover the weight importance of each attribute, including both intrinsic and extrinsic. When this phase is done, cues such as freshness, taste, juiciness and flavor can be evaluated by a trained sensory panel. For this purpose, a five-level quality scoring method can be used. Furthermore, a desirable price can be estimated by consumers, using a hedonic scale. Other characteristics from Table 4 andTable 5 can be instrumentally or scale-based evaluated, such as meat color, cut, marbling, amount of drip, fat content and texture. The color values can be determined using a colorimeter (e.g., CR-400 Chroma Meter), spectrophotometer (e.g., Minolta CM-600d), Near-Infrared Spectroscopy, etc. Furthermore, coupling methods such as spectroscopy and imaging techniques and marbling can be examined. The cut of meat and marbling can be evaluated using scale-based methods and meat standards. Finally, for fat content evaluation and texture measurements, it has been proposed to use a food scanner and texture analyzer (texture profile analyses or WBSF), respectively. As it has been mentioned earlier in this paper, the amount of drip can be measured using the parameter of WHC. The difference between attributes presented in Table 3, Table 4 and Table 5 is that the latter are supposed to be included in the QI equation. The quality cues in Table 3 were presented in the role of meat quality factors.

It can be noticed that some extrinsic characteristics cannot be used directly in the QI formula but can be used as factors. In that context, the remaining characteristics that are not presented in these tables but in wheels can be involved in making appropriate environments for using the QI, such as different brands, sellers, animal welfare programs, breeds, feeding systems, processing technologies and so on and can be used to differentiate samples. Furthermore, the type of package, storage temperature and level of hygiene can be used as determinants that can influence the quality of a product during storage time [13].

One of the first examples of linking price with the QI is in the study by Finotti et al. [10]. When it comes to the maturation of the meat, this attribute is in the first group of characteristics rather than in third one, because a longer aging process leads to water loss and more tender and flavor-changed meat. This characteristic is related to the process of meat aging and can be expressed in days. Thus, wet aging can last between three and 83 days, while dry aging requires several weeks [71]. 

## 6. Conclusions and Future Trends

This review outlines the importance of understanding intrinsic and extrinsic meat quality cues and the potential of using meat quality wheels. It has been elaborated why quality wheels may be useful tools for both consumers and meat producers in finding the optimal number of quality characteristics that are considered at the point of meat purchase. According to previous literature, meat color, sensory characteristics and fat content have been shown as the most important intrinsic quality cues of fresh beef and pork from the consumer’s point of view, where odor, tenderness, juiciness, flavor and overall liking, respectively, were the most frequently examined. On the other side, when it comes to extrinsic quality cues, price, certification logos and brand name were noted as the most important for evaluating beef quality. Furthermore, price, breed, animal welfare and a veterinary certificate were noted as main determinants of pork quality. As an outcome, this review has identified approaches in evaluating individual quality characteristics and metrics for developing an overall meat quality index. The lack of some characteristics in previous literature, such as experience attributes for the extrinsic quality of beef and credence attributes for the intrinsic quality of pork, is a limitation of this study, and this aspect may represent a theme for further research. Future research should focus on validating the proposed meat quality index for both beef and pork meat employing consumer research. The proposed QI formula does not pretend to be the unique practical answer to the need for evaluating quality in the meat industry, but seeks to show a way through which the base of the quality evaluation can be established. The main feature of this index is its flexibility, because it can be adapted to every choice of the quality parameters presented in this study. Hence, both intrinsic and extrinsic characteristics are applicable for this type of quality index. For the purpose of a validation process in future research, we have proposed quality parameters, how to group them and the mathematical index in terms of the chosen parameters.

## Figures and Tables

**Table 1:** Three phases of search for the literature on beef quality.

	Initial Database		Google Scholar		
First phase of search	Search field		Abstract, Title, keywords		
	Keywords		Intrinsic and extrinsic quality characteristics of beef		
	Search settings		Use all words, Sort by importance and best matching with keywords		
	Period		2018–2021		
	Number of publications		*n* = 9480		
	Additional keywords		Intrinsic and extrinsic quality characteristics of beef consumer preference		
	Number of publications		*n* = 4020		
**Second phase of search**	Additional criteria 1 (only full-text articles)		Thesis and chapters excluded		
	Additional criteria 2 (subject is fresh beef quality)		Publications excluded (subject is carcass quality, quality of frozen beef, beef products)		
	Additional criteria 3 (subjects are intrinsic and extrinsic characteristics of product)		Publications excluded (subjects are intrinsic and extrinsic factors in meat production)		
	Additional criteria 4 (consumer’s preference)		Publications excluded (production perspective)		
Third phase of search	Total number of articles retained		*n* = 41		
	Databases included		Google Scholar, Science Direct, Wiley online library, Emerald Insight, MDPI, Frontiers		
Google Scholar	Science Direct	Wiley online library	Emerald Insight	MDPI	Frontiers
13	14	2	1	10	1
Research articles			Review articles		
35			6		

**Table 2:** Three phases of search for the literature on pork quality.

	Initial Database		Google Scholar	
First phase of search	Search field		Abstract, Title, keywords	
	Keywords		Intrinsic and extrinsic quality characteristics of pork	
	Search settings		Use all words, sort by importance and best matching with keywords	
	Period		2018–2021	
	Number of publications		*n* = 9220	
	Additional keywords		Intrinsic and extrinsic quality characteristics of pork consumer preference	
	Number of publications		*n* = 2690	
Second phase of search	Additional criteria 1 (only full-text articles)		Thesis and chapters excluded	
	Additional criteria 2 (articles are about pork quality)		Publications excluded (subject is carcass quality, quality of frozen pork, pork products)	
	Additional criteria 3 (subjects are the intrinsic and extrinsic characteristics of the product)		Publications excluded (subjects are intrinsic and extrinsic factors in meat production)	
	Additional criteria 4 (consumer’s preference)		Publications excluded (production perspective)	
Third phase of search	Total number of articles retained		*n* = 15	
	Databases included		Google Scholar, Science Direct, Wiley online library, Emerald Insight, MDPI, Frontiers	
Google Scholar	Science Direct	Elsevier	MDPI	IOP Conference Series
2	4	2	6	1
Research articles			Review articles	
12			3	

**Table 3:** Suggested characteristics for assessing different quality of meat and meat products.

Meat or Meat Product	Characteristics	Purpose	Key Quality and Safety Terms	Reference
Minced pork meat	Sensory analysis, color and oxidation measurements	New package	Sensory quality Meat quality	[63]
Australian beef loins	pH, color, weight loss during ageing, retail yield, total water content, myofibrillar fragmentation index and lipid (TBARS) and protein (total carbonyl content) oxidation	Different ageing methods	Eating quality of beef loins using the Meat Standards Australia (MSA) sensory protocols	[64]
Beef and chicken meats	Microorganisms, amino acid composition profile, chemical composition, mineral concentrations, water mobility and fat content	The effects of repeated freeze–thaw cycles	Meat quality Hygienic quality Stable quality	[65]
Beef	pH, color, shear force and cooking loss, water-holding capacity and the glycolytic potential	The occurrence of DFD beef	Meat quality	[66]
Beef loins	pH, color, purge, cooking loss, shear force, sarcomere length, particle size and sensory analysis	The prediction of meat and eating quality traits	Sensory quality Meat quality	[67]
Pork	Purine measurements and sensory analysis	The effect of purine content	Sensory quality	[68]
Pork	pH and redox potential	The effect of different types of electrolyzed water	Microbiological and oxidative quality	[69]
Beef	pH, smell, weight loss, water holding capacity, shear force and consumer preference	Different package	Meat quality Microbial quality Consumer test	[70]

**Table 4:** Categorization of characteristics emerged from quality wheel for beef.

	Nearer to the Target Value Is Better	A Smaller Characteristic’s Value: Better Quality	A Higher Characteristic’s Value Is Better
**Intrinsic attributes**			
**Credence attributes**			
Nutritional values of vitamin B_12_, Zinc, Iron, so on.			x
**Experience attributes**			
Freshness			x
Taste			x
Tenderness	x		
Juiciness	x		
Odor	x		
Flavor	x		
**Search attributes**			
Meat color		x	
Cut	x		
Marbling	x		
Amount of drip		x	
Texture	x		
Hedonic/preference evaluation			x
**Extrinsic attributes**			
Price		x	
Hedonic/preference evaluation			x

* Where x stands for labeling the group (column) where certain attribute belongs to.

**Table 5:** Categorization of characteristics emerged from quality wheel for pork.

	Nearer to the Target Value Is Better	A Smaller Characteristic’s Value: Better Quality	A Higher Characteristic’s Value Is Better
**Intrinsic attributes**			
**Experience attributes**			
Flavor	x		
Tenderness	x		
Taste			x
Juiciness	x		
Leanness	x		
**Search attributes**			
Cut	x		
Fat content		x	
Meat color		x	
Marbling	x		
Overall appearance			x
Amount of drip		x	
Texture	x		
Hedonic/preference evaluation			x
**Extrinsic attributes**			
Price		x	
Maturation of the meat	x		
Hedonic/preference evaluation			x

* Where x stands for labeling the group (column) where certain attribute belongs to.

## Data Availability

The data used to support the findings of this study are included within the article.
